# Calcineurin activation causes retinal ganglion cell degeneration

**Published:** 2012-11-29

**Authors:** Juan Qu, Roland Matsouaka, Rebecca A. Betensky, Bradley T. Hyman, Cynthia L. Grosskreutz

**Affiliations:** 1 Massachusetts Eye and Ear Infirmary, Department of Ophthalmology, Harvard Medical School, Boston, MA; 2 Department of Biostatistics, Harvard School of Public Health, Boston, MA; 3 Department of Neurology, Massachusetts General Hospital, Boston, MA

## Abstract

**Purpose:**

We previously reported that calcineurin, a Ca^2+^/calmodulin-dependent serine/threonine phosphatase, is activated and proposed that it participates in retinal ganglion cell (RGC) apoptosis in two rodent ocular hypertension models. In this study, we tested whether calcineurin activation by itself, even in the absence of ocular hypertension, is sufficient to cause RGC degeneration.

**Methods:**

We compared RGC and optic nerve morphology after adeno-associated virus serotype 2 (AAV2)–mediated transduction of RGCs with constitutively active calcineurin (CaNCA) or unactivated, wild-type calcineurin (CaNwt). Retinas and optic nerves were harvested 7–16 weeks after injection of the AAV into mouse vitreous. In flatmounted retinas, the transduced RGCs were identified with immunohistochemistry. The morphology of the RGCs was revealed by immunostaining for neurofilament SMI32 or by using GFP-M transgenic mice. A modified Sholl analysis was applied to analyze the RGC dendritic morphology. Optic nerve damage was assessed with optic nerve grading according to the Morrison standard.

**Results:**

CaNwt and CaNCA were highly expressed in the injected eyes. Compared to the CaNwt-expressing RGCs, the CaNCA-expressing RGCs had smaller somas, smaller dendritic field areas, shorter total dendrite lengths, and simpler dendritic branching patterns. At 16 weeks, the CaNCA-expressing eyes had greater optic nerve damage than the CaNwt-expressing eyes.

**Conclusions:**

Calcineurin activation is sufficient to cause RGC dendritic degeneration and optic nerve damage. These data support the hypothesis that calcineurin activation is an important mediator of RGC degeneration, and are consistent with the hypothesis that calcineurin activation may contribute to RGC neurodegeneration in glaucoma.

## Introduction

Glaucoma is a chronic neurodegenerative disease in which retinal ganglion cells (RGCs) degenerate, leading to gradual vision loss and ultimately blindness. RGC death is commonly, but not always, associated with elevated intraocular pressure (IOP). Conditions such as neurotrophin deprivation, glial activation, ischemia, oxidative stress, and excitotoxicity have been suggested to play a role in glaucoma (reviewed in [1]). Ultimately, RGCs appear to die by apoptosis. Calcineurin is a Ca^2+^/calmodulin-dependent serine/threonine phosphatase. It is widely expressed in mammalian tissues including the retina [2,3], brain [4,5], and immune cells [6]. In neurons, calcineurin is involved in morphological neurodegeneration [7] and apoptosis [8-11]. We recently suggested that activation of calcineurin acts as a key initiating step of apoptotic pathways in RGCs in mouse and rat models of elevated IOP. This hypothesis was based on observations that a constitutively active truncated form of calcineurin was present in these models, and blocking calcineurin with a pharmacological inhibitor, FK506, was neuroprotective [12]. However, FK506 has some off-target effects [13], so it remains formally possible that FK506 protection is not due to inhibition of calcineurin. To further test the model that calcineurin activation is a critical component of neurodegenerative cascades in RGCs, we tested the prediction that activating calcineurin, even without elevated intraocular pressure, would lead to a phenotype of RGC degeneration similar to that seen in models of glaucoma.

In a primate model of glaucoma, retinal parasol cells from glaucomatous eyes had a smaller soma, a smaller and less complex dendritic arbor, and a shorter total dendrite length [14]. In DBA/2J mice, a mouse strain that spontaneously develops glaucoma, dendritic degeneration and somal shrinkage precede RGC death [15]. In addition, there is progressive RGC axon loss in the optic nerve as glaucoma progresses [16]. In the mouse and rat experimental glaucoma models, calcineurin is activated in glaucomatous eyes as judged by the presence of truncated constitutively activated calcineurin [12]. In this study, we tested the hypothesis that calcineurin activation causes RGC somal, dendritic, and axonal degeneration similar to glaucomatous RGC degeneration.

**Figure 1:**
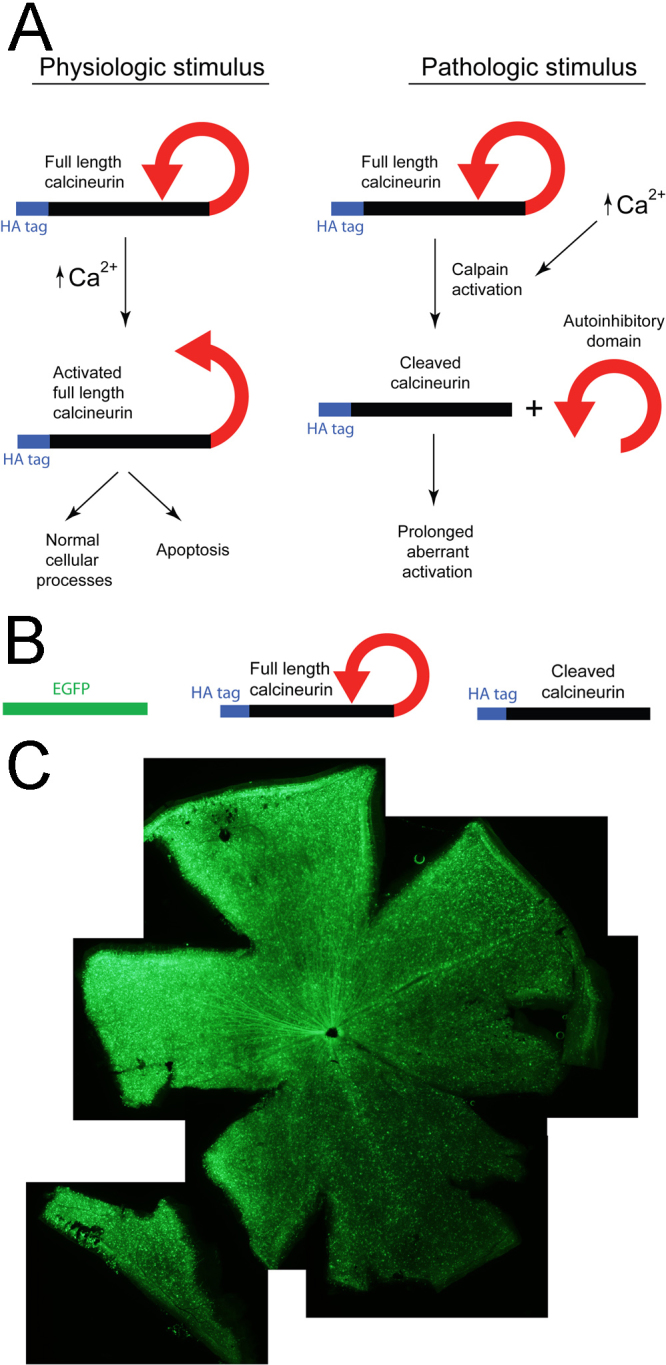
AAV2-mediated transduction was used to study calcineurin function in mouse retinal ganglion cells (RGCs). **A**: Calcineurin can be activated under physiologic conditions or pathological conditions [8]. Under physiologic conditions, binding of Ca^2+^/calmodulin reversibly exposes the catalytic domain and activates the enzyme. Under pathological conditions, cleavage of the autoinhibitory domain leaves the catalytic domain constitutively active. **B**: Three AAVs were used to express EGFP (control), HA-tagged full-length calcineurin (CaNwt, unactivated), and HA-tagged cleaved calcineurin (CaNCA, constitutively active) in RGCs. **C**: EGFP was expressed in RGCs throughout the retina nine weeks after intravitreal injection of AAV-EGFP.

Wild-type calcineurin contains a catalytic domain and an autoinhibitory domain. Under physiologic conditions, the autoinhibitory domain blocks the catalytic domain and inhibits enzyme activity in the absence of Ca^2+^/calmodulin. Upon binding of Ca^2+^/calmodulin, calcineurin undergoes a conformational change, exposes the catalytic domain, and activates the enzyme [4,17]. Under pathological conditions, the autoinhibitory domain can be cleaved by proteases such as calpain [8,18], leaving the catalytic domain constitutively active (Figure 1A). We used adeno-associated virus serotype 2 (AAV2) to deliver wild-type calcineurin and constitutively active (C-terminal autoinhibitory domain truncated [7]) calcineurin to RGCs in vivo. We found that constitutively active calcineurin caused more RGC morphological degeneration and optic nerve damage than wild-type calcineurin, which did not differ from control injections of AAV2. These data suggest that calcineurin activation is a critical mediator of RGC degeneration, and are consistent with the hypothesis that calcineurin activation in models of glaucoma is an important part of the cascade of events that lead to RGC degeneration.

## Methods

### Animals

All experiments were performed in accordance with the ARVO Statement for the Use of Animals in Ophthalmic and Vision Research. In human patients and in spontaneous animal models [14-16], glaucoma is more prevalent in an aged population. To make our results comparable, we conducted our studies on nine- to 12-month-old female C57BL/6 mice (Charles River Laboratories, Boston, MA) and 12-month-old female GFP-M mice (Strain name Tg(Thy1-EGFP)MJrs/J, The Jackson Laboratory, Bar Harbor, ME). Similar to the Thy1-YFP mice used in a previous RGC morphology study [19], in GFP-M mice a small proportion of RGCs express green fluorescent protein (GFP) in their somas, axons, and dendrites [20]. The sparse labeling of RGCs avoids overlap of dendrites of neighboring labeled cells, thus allowing clear visualization of the entire dendritic arbor in each individual RGC.

### Intravitreal injection

AAV serotype 2 was used to express calcineurin in RGCs because the virus primarily transduces RGCs when administrated intravitreally. Three AAVs carrying 1) enhanced green fluorescent protein (EGFP), 2) hemagglutinin (HA)-tagged mouse wild-type calcineurin (CaNwt), and 3) an HA-tagged constitutively active truncated form of calcineurin (CaNCA) were used in this study (Figure 1B). The detailed structures of the AAV vectors were previously described by Wu and colleagues [7]. Briefly, EGFP, HA-CaNwt, and HA-CaNCA (amino acid residues 1–399) were cloned into rAAV-cytomegalovirus immediate-early enhancer/chicken beta-actin promoter-woodchuck posttranscriptional regulatory element vector. High-titer AAV2 was produced using plasmid triple transfection. The expression of EGFP was readily visible under a confocal microscope. The expression of CaNwt and CaNCA was revealed with immunohistochemistry using an antibody against their N-terminal HA tag. All three AAVs had similar transduction rates.

Mice were anesthetized with a mixture of ketamine (100 mg/kg) and xylazine (10 mg/kg) (both from Webster Veterinary Supply, Sterling, MA). One drop of 0.5% proparacaine was applied topically for local anesthesia. Using a glass needle (tip diameter about 50 μm), 1 μl of virus (titer, 2×10^12^ viral genomes/ml) was injected into the vitreous at about 1 mm behind the limbus. AAV2 is known to have a relative tropism for RGCs in the retina. After the injection, antibiotic ointment was applied, and the mice recovered from anesthesia on a heating pad.

### Tissue harvesting

Mice were overdosed with a mixture of ketamine and xylazine (i.p. 300 mg/kg and 30 mg/kg, respectively), the eyes were enucleated, and the mice were euthanized immediately by cervical dislocation. The retinas were dissected in PBS (8.06 mM Na_2_HPO_4_, 1.94 mM KH_2_PO_4_, 2.7 mM KCl, 137 mM NaCl), flatmounted on filter paper and fixed in 4% paraformaldehyde for 2 h, washed in PBS and stored at 4 °C. The optic nerves were fixed in 2.5% glutaraldehyde and 2% formaldehyde in 0.1 M cacodylate buffer with 0.08 M CaCl_2_ at 4 °C overnight, washed in 0.1 M cacodylate buffer, and stored at 4 °C.

### Immunohistochemistry

Flatmounted retinas were blocked in 4% normal goat serum (Invitrogen, Eugene, OR) in PBS at 4 °C overnight, incubated in rabbit anti-HA (1:200, Invitrogen) and/or mouse anti-SMI32 (1:100, Covance, Emeryville, CA) at 4 °C for 1 week, rinsed in PBS, and incubated in Alexa 488 or 594 conjugated goat antirabbit (1:250, Invitrogen) and/or goat antimouse (1:500, Invitrogen) antibodies at 4 °C for 1 week, rinsed in PBS, and mounted with Prolong Gold antifade reagent (Invitrogen). Images were taken with a Bio-Rad Radiance laser scanning confocal microscope (Hercules, CA) and edited in ImageJ (National Institutes of Health, Bethesda, MD).

### Optic nerve grading

The optic nerve was post-fixed for 1.5 h in 2% aqueous OsO_4_, dehydrated in graded ethanols, transitioned in propylene oxide, infiltrated with propylene oxide and epon mixtures (Tepon resin, Tousimis, Rockville, MD), embedded in epon, and cured for 24–48 h at 60 °C. One-micron sections were cut in a plane perpendicular to the axis of the nerve on a Leica Ultracut UCT (Buffalo Grove, IL) and stained with 1% toluidine blue in 1% borate buffer. Images tiling the entire optic nerve were taken with an Olympus BX51 microscope (Albertslund, Denmark). Images from the experimental eyes and control eyes were masked, randomly mixed, and then graded by four well-trained observers using the Morrison standard [21]. The average grades from the four observers were taken as the grades of the optic nerves.

### Morphological analysis

The right eye and the left eye of three 12-month-old GFP-M mice were injected with AAVs carrying CaNwt and CaNCA, respectively. The mice were sacrificed 16 weeks after the injection, and the retinas were flatmounted. Image stacks of GFP-expressing RGCs were taken with a Bio-Rad Radiance laser scanning confocal microscope. The z-projections of the image stacks were manually traced in an ImageJ plugin NeuronJ. The tracings were then used for all the morphological analyses. In total, there were 23 CaNwt/GFP double-labeled RGCs, and 31 CaNCA/GFP double-labeled RGCs with a well-defined dendritic arbor. The distribution was even, and we did not see a significant difference among the RGCs collected from the three mice. Therefore, the data were combined for the morphological analyses except the Wilcoxon rank-sum test, where the permutations were performed within each mouse.

The total length of the RGC dendrites was measured in NeuronJ. The areas of the RGC soma and dendritic fields were measured using a custom ImageJ macro. Sholl analysis was performed to evaluate the complexity of the RGC dendrites using the same macro. For each RGC, Sholl analysis was performed by counting the number of dendrite intersections with concentric circles of gradually increasing radius centered on the centroid of the soma. The number of intersections was plotted against the radius, and the area under the curve (AUC) was calculated using the trapezoidal method. We used the AUC as we had hypothesized that it would be a powerful test statistic for distinguishing between the two conditions that achieved 0 intersections at similar radii, but for which one had consistently more intersections at a set of smaller radii. The Wilcoxon rank-sum test was then used to compare the AUCs for the CaNwt and CaNCA conditions. Because each mouse contributed several AUCs to each condition, we could not use the standard p value calculation for the Wilcoxon test that assumes independence among observations. Instead, we calculated the p value based on a permutation approach. W_o_ was the Wilcoxon test statistic comparing CaNwt and CaNCA for the original data set. W_perm_ was the Wilcoxon test statistic for permuted data; within each mouse, we permuted the labels of CaNwt and CaNCA, keeping the Sholl plots for each condition intact and the number of each condition for each mouse fixed. For each permutation, we calculated a Wilcoxon test statistic. The permutations were repeated 2,000 times to obtain a null permutation distribution of the Wilcoxon test statistics, to which we compared the actual obtained value, W_o_. The permutation p value is the estimated probability of W_perm_ > W_o_ and represents the probability of observing the value that we observed under the null hypothesis of no difference between conditions. The null hypothesis was rejected when the permutation p value <0.05. Although W_o_ and W_perm_ were calculated on RGCs pooled from all the mice, the permutation was performed independently within each mouse, a more conservative estimate since the RGCs from the left eye and the right eye of an individual mouse may be more alike than the RGC characteristics in retinas from different mice.

## Results

### Calcineurin activation caused neurofilament degeneration in retinal ganglion cell dendrites

AAVs carrying HA-tagged mouse wild-type calcineurin (CaNwt) or an HA-tagged truncated form of calcineurin (CaNCA) were injected intravitreally into one eye of the C57Bl/6 mice. The other eye was injected with an AAV carrying EGFP as a control. The wild-type calcineurin (CaNwt) contains the catalytic domain and the inhibitory domain; therefore, CaNwt activity is under biologic control. In contrast, the truncated form of calcineurin (CaNCA) does not contain the autoinhibitory domain and is constitutively active (Figure 1B). As shown in Figure 1C, EGFP was expressed in retinal ganglion cells throughout the retina. CaNwt and CaNCA had similar expression patterns, revealed by immunostaining against their HA tag (data not shown).

**Figure 2:**
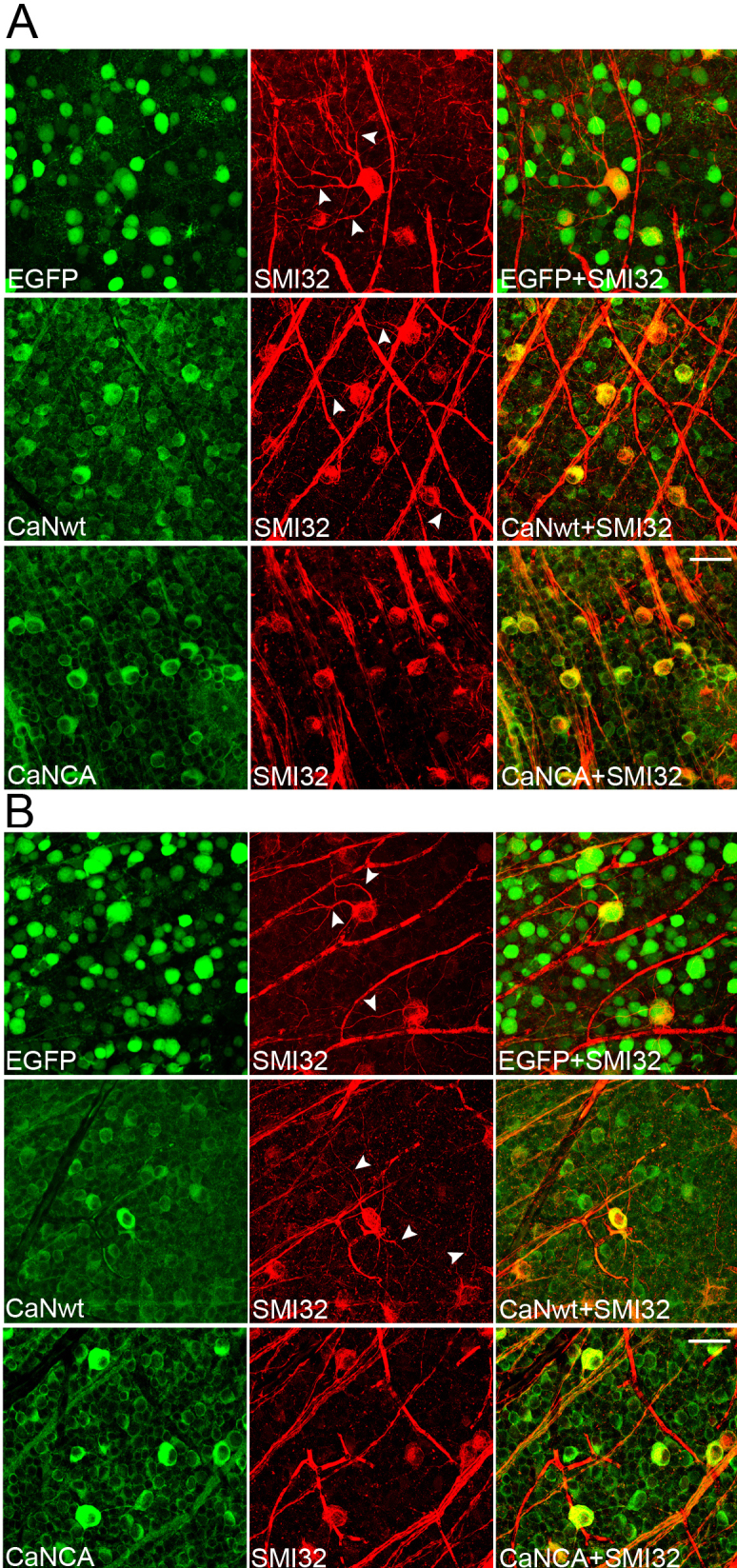
Calcineurin activation caused neurofilament degeneration in retinal ganglion cell (RGC) dendrites. C57BL/6 mice were intravitreally injected with AAV carrying EGFP (control), HA-tagged full-length calcineurin (CaNwt), and HA-tagged cleaved calcineurin (CaNCA). The expression of transduced CaNwt and CaNCA was revealed by the antibody against their HA tag. RGC dendritic morphology was assessed with immunohistochemistry against SMI32. Seven weeks (**A**) and sixteen weeks (**B**) after injection, SMI32-IR was reduced in RGC dendrites in the CaNCA-expressing retinas at both time points, but not in the EGFP- or CaNwt-expressing retinas. The arrowheads point at the dendrites in EGFP- or CaNwt-expressing RGCs. No consistent difference was observed in SMI32-IR in RGC somas and axons. Scale bars are 50 µm.

Immunohistochemistry against neurofilament SMI32 labels a subpopulation of RGCs in the retina [15]. SMI32 immunoreactivity (SMI32-IR) is detectable in RGC somas, dendrites, and axons. Seven weeks after AAV injection, compared to the EGFP-expressing retinas, SMI32-IR was clearly reduced in the RGC dendrites, especially in the higher-order dendrites, in the CaNCA-expressing retinas (six mice). The SMI32-IR in RGC dendrites in the CaNwt-expressing retinas (six mice) was still extensive and was not different from the control EGFP-expressing retinas (Figure 2A). The SMI32-IR in RGC somas and axons did not show a consistent change either in the CaNwt-expressing retinas or in the CaNwt-expressing retinas. Results were similar 16 weeks after AAV injection (six mice for CaNwt/EGFP and six mice for CaNCA/EGFP) (Figure 2B). The reduction in SMI32-IR in the RGC dendrites indicated degradation of the neurofilaments, and suggested that calcineurin activation could cause degeneration of RGC dendrites.

### Calcineurin activation caused degeneration of retinal ganglion cell somas and dendrites

**Figure 3:**
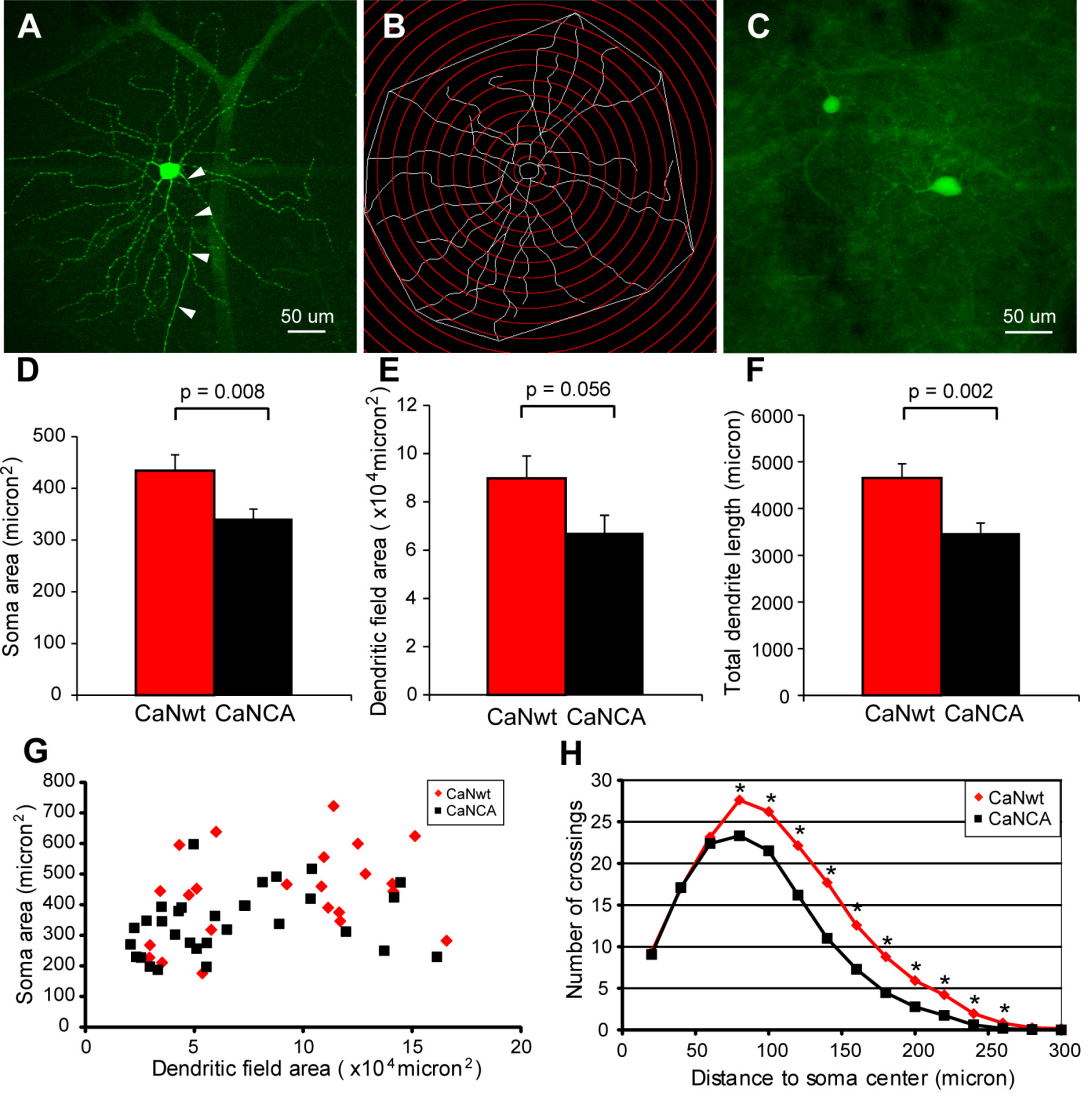
Calcineurin activation caused retinal ganglion cell (RGC) somal shrinkage and dendritic degeneration. GFP-M mice were intravitreally injected with AAV carrying full-length calcineurin (CaNwt) in one eye and AAV carrying cleaved calcineurin (CaNCA) in the other eye. **A**: A sample GFP-expressing RGC displayed well labeled dendrites. Arrowheads point at the axon. **B**: The RGC in (**A**) was digitally traced in NeuronJ. Soma area, dendritic field area, and total dendrite length were measured. Sholl analysis was applied. **C**: A few RGCs had GFP-expressing soma, but the higher-order dendrites or the entire dendritic tree were not visible. These RGCs were rare, but more in the CaNCA-expressing retinas. They were not included in the morphological analysis. **D**: RGCs had smaller soma areas in the CaNCA-expressing retinas. (All results were mean±standard error of the mean, two-tailed unpaired Student *t* test). **E**: RGCs had smaller dendritic field areas in the CaNCA-expressing retinas. **F**: RGCs had shorter total dendrite length in the CaNCA expressing retinas. **G**: The soma areas of all the RGCs were plotted against the dendritic field areas. The CaNCA-expressing RGCs cluster in the lower-left corner, which indicates they had smaller soma areas and smaller dendritic field areas. **H**: Sholl analysis showed fewer RGC dendritic branches were present in CaNCA-expressing retinas. (*p<0.05, two-tailed unpaired Student *t* test).

Although immunohistochemistry of SMI32 is commonly used to study RGC morphology, it has certain limitations. The antibody against SMI32 labels only the nonphosphorylated form of neurofilament H; therefore, the phosphorylation state can affect the SMI32-IR. In addition, the overlap of the dendrites from neighboring SMI32-positive RGCs interferes with analysis of higher-order dendrites. To assess whether the loss of SMI32-IR reflected a loss of dendritic structure or just a change in neurofilament phosphorylation, and to evaluate the entire dendritic arbor more precisely, we repeated the experiment using GFP-M transgenic mice [20] for morphological analysis. In the retina of GFP-M mice, a small proportion of RGCs express GFP, allowing visualization of the entire dendritic arbor (Figure 3A) in those cells. AAVs carrying CaNwt and CaNCA were injected into the right eye and the left eye, respectively, of the GFP-M mice. These GFP-expressing RGCs were imaged and manually traced if (1) there was a clear axon confirming the identity of the cell as an RGC and (2) the soma contained HA immunoreactivity indicating that the RGC had been transduced with the AAV. The digital tracings were used for all the following quantitative morphological analyses (Figure 3B).

Sixteen weeks after AAV injection, compared to the CaNwt-expressing RGCs (n=23), the CaNCA-expressing RGCs (n=31) had smaller somas (Figure 3D; CaNCA 342±18 µm^2^ versus CaNwt 434±31 µm^2^, p=0.008; all results are mean±standard error, two-tailed unpaired Student *t* test) and smaller dendritic field areas (Figure 3E; CaNCA 67209±7257 µm^2^ versus CaNwt 89805±9203 µm^2^, p=0.056). In the CaNwt-expressing RGCs and the CaNCA-expressing RGCs, there was a direct correlation between the size of the RGC soma and the size of the dendritic field, i.e., RGCs with smaller somas usually had smaller dendritic fields, and RGCs with larger somas usually had larger dendritic fields (Figure 3G). However, although the CaNwt-expressing RGCs had relatively even distribution among all sizes, the CaNCA-expressing RGCs clustered in the lower left corner of the graph (Figure 3G), suggesting they had smaller somas and smaller dendritic fields.

Sholl analysis was applied on the RGC tracings to quantitatively evaluate the effects of calcineurin activation on the complexity of the RGC dendrites. The analysis was performed by counting the number of dendrite intersections with concentric circles of gradually increasing radius centered on the centroid of the soma (Figure 3B). As shown in Figure 3H, there was no difference in the number of intersections between the CaNwt- and CaNCA-expressing RGCs within 60 µm to the soma centroid, but there were significantly fewer intersections in the CaNCA-expressing RGCs 80 µm and further from the soma. This indicated that although the number of primary dendrites had not been affected at 16 weeks, the number of higher-order dendritic branches was significantly reduced by calcineurin activation. To capture the overall complexity of the RGC dendrites, we used a novel AUC approach to analyze the data, followed by a Wilcoxon rank-sum test to assess significance (see the Methods section for details). The permutation p value of 0.012 indicates that there is a statistically significant difference between CaNCA-expressing RGCs compared to CaNwt-expressing RGCs, with CaNCA leading to a substantial decrease in dendritic complexity. Another measure of dendritic structure was also employed: adding the length of all the dendritic segments together. The CaNCA-expressing RGCs had significantly shorter total dendrite lengths (Figure 3F; CaNCA 3473±216 µm versus CaNwt 4652±306 µm, p=0.002). This is likely due to the shrinkage of higher-order dendrites, consistent with the Sholl analysis results.

All the HA-positive RGCs with clearly GFP-labeled dendrites were traced and included in the analyses in the AAV-injected GFP-M mice (Figure 3A). However, rare RGCs expressed GFP in the somas, but their dendrites, especially the higher-order dendrites, were invisible (Figure 3C). These RGCs were excluded from the morphological analyses. In our experience working with GFP-M mice (young and old), well-labeled RGCs show clear endings of the terminal dendrites, i.e., the dendrite terminals end abruptly instead of gradually fading away. In the case when an RGC weakly expresses GFP, the soma (where the GFP concentrates) still shows up, but the dendritic tree can range from faint to invisible. When immunohistochemistry against GFP is performed, some of these faint/invisible dendrites can become visible. Therefore, the lack of clear dendrites could indicate complete degeneration of the dendrites, but could also be due to the low expression level of GFP. Because these cells are more often encountered in CaNCA-expressing retinas than in CaNwt-expressing retinas, by excluding them, the statistically significant effects of calcineurin activation on RGC morphological degeneration reported above could actually be an underestimation.

### Calcineurin activation caused optic nerve damage

**Figure 4:**
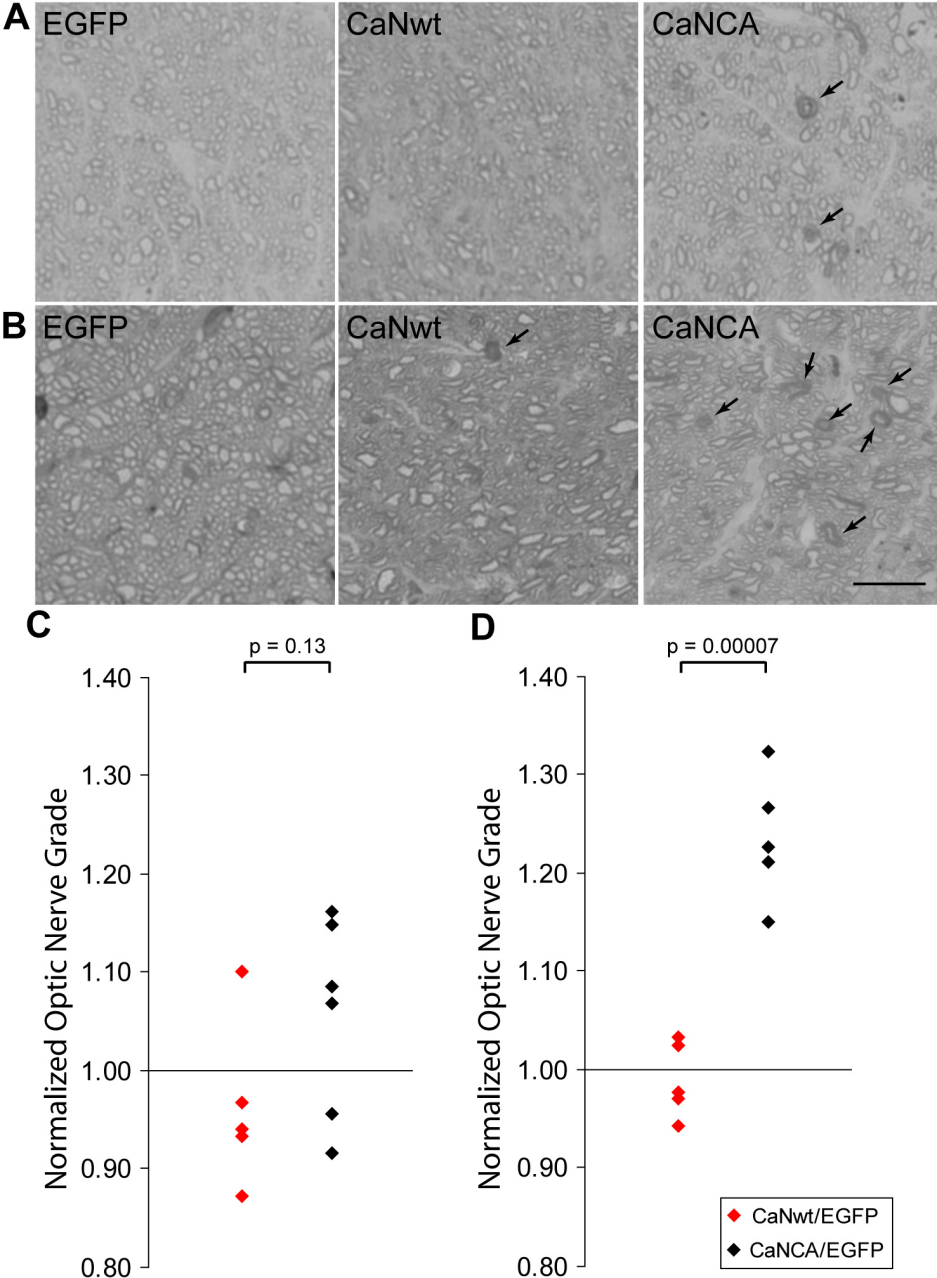
Prolonged calcineurin activation caused optic nerve damage. **A** and **B**: Sample optic nerve images showed healthy and degenerated axons seven weeks (A) and 16 weeks (B) after AAV injection. Arrows point at degenerated retinal ganglion cell axons. Scale bar is 10 µm. **C** and **D**: The optic nerve degeneration was assessed by optic nerve grading using the Morrison standard. Higher grades were assigned to more damaged optic nerves. The optic nerve grades of the full-length calcineurin (CaNwt)-expressing eyes and the cleaved calcineurin (CaNCA)-expressing eyes were normalized to their contralateral control EGFP-expressing eyes. The optic nerves in the CaNCA-expressing eyes were not different from the CaNwt-expressing eyes seven weeks after AAV injection (**C**), but were more damaged 16 weeks after AAV injection (**D**; two-tailed unpaired Student *t* test).

The axons of RGCs bundle together and form the optic nerve. In glaucoma, these axons degenerate as the disease progresses [16]. We found that extended activation of calcineurin also caused RGC axon degeneration in the optic nerve. In C57BL/6 mice seven weeks after intravitreal injection of AAVs carrying EGFP, CaNwt, or CaNCA, the optic nerves from the EGFP-expressing eyes and the CaNwt-expressing eyes were morphologically normal, while the optic nerves from the CaNCA-expressing eyes had some degenerating axons (Figure 4A). Sixteen weeks after AAV injection, the optic nerves from the EGFP-expressing eyes and the CaNwt-expressing eyes still looked healthy, but the optic nerves from the CaNCA-expressing eyes had notably more degenerating axons (Figure 4B).

To quantitatively evaluate the condition of the optic nerves, we adapted a grading system from the Morrison standard [21]. The images of the optic nerves were masked and graded by four trained observers, and the average was taken as the grade of each optic nerve. Healthy optic nerves received lower grades while more degenerated optic nerves received higher grades. The grades of the CaNwt-expressing eyes (five mice, seven weeks; five mice, 16 weeks) and the grades of the CaNCA-expressing eyes (six mice, seven weeks; five mice, 16 weeks) were normalized to the grades of their contralateral EGFP-expressing eyes. There was no significant difference between the CaNwt- and CaNCA- expressing eyes at seven weeks (two-tailed unpaired Student *t* test, p=0.13), but the grades of the CaNCA-expressing eyes were significantly higher 16 weeks after injection (p=0.00007; Figure 4C, D). This indicated that prolonged activation of calcineurin caused optic nerve damage.

Neurofilament degradation in RGC dendrites was already apparent seven weeks after AAV injection, while axon degeneration did not become significant (at least at the level of our observation) until much later. This suggested that calcineurin-caused RGC damage was likely to originate from the soma, and it might take longer for the effects to reach the axons in the optic nerve.

## Discussion

### Calcineurin activation is an important mediator of retinal ganglion cell death in glaucoma

We previously demonstrated that calcineurin is activated in response to ocular hypertension in rat and mouse models of experimental glaucoma [12]. Activated calcineurin dephosphorylates Bcl-2-associated death promoter protein (BAD) and promotes cytochrome *c* release and RGC death [12]. As a Ca^2+^/calmodulin-dependent phosphatase, calcineurin can be directly activated by elevated intracellular calcium concentration. In glaucoma, many insults such as excitotoxicity [22], ischemia [23,24], and activation of the transient receptor potential cation channel, subfamily V, member 1 (TRPV1) [25] can lead to an increase in intracellular calcium concentration and therefore activate calcineurin. The increase in intracellular calcium can also activate Ca^2+^-dependent proteases such as calpain, which cleaves off the autoinhibitory domain of calcineurin and causes prolonged irreversible activation of calcineurin [7]. Inhibitors of calpain are neuroprotective in experimental glaucoma [26-28]. We have shown that calpain-mediated cleavage of calcineurin occurs in experimental glaucoma [8] and that inhibitors of calcineurin [29,30] are effective neuroprotective agents in experimental glaucoma. Our data in this study show that even in the absence of ocular hypertension, calcineurin activation itself can cause RGC somal shrinkage and dendritic degeneration (Figure 2 and Figure 3), and optic nerve damage (Figure 4), similar to the RGC degeneration that happens in glaucomatous DBA/2J mice [15,16] and in primate models [14]. This further supports our hypothesis that calcineurin activation is an important mediator of RGC death in glaucoma.

### Retinal ganglion cell degeneration is not restricted to one or a few retinal ganglion cell subtypes

More than a dozen RGC subtypes are present in the mouse retina [31-33]. Although some studies indicate that large RGCs are more vulnerable than small RGCs in human glaucoma [34,35], no subtypes of RGCs have been found to be especially vulnerable or resistant to degeneration in DBA/2J mice [15]. This could be due to differences among species, but it is also possible that RGC shrinkage is a generalized response in glaucoma leading to the impression of the disappearance of large RGCs and the persistence of small RGCs observed in earlier studies.

The latter interpretation is supported by a recent long-term in vivo imaging study. Leung and colleagues serially recorded the morphological degeneration of 125 RGCs for six months after optic nerve crush using a confocal scanning laser ophthalmoscope [19]. The researchers found that essentially all RGCs degenerate after optic nerve crush. The RGC degeneration begins with dendritic shrinkage and is followed by somal and axonal loss. This agrees well with our observations on RGC degeneration induced by calcineurin activation. Dendritic degeneration was already apparent seven weeks after AAV injection, but axonal degeneration could not be detected using our current techniques until much later. In Figure 3G, compared to the CaNwt-expressing RGCs, the distribution of the CaNCA-expressing RGCs appeared to shift to the lower-left corner of the graph, supporting the hypothesis that all RGCs degenerate and shrink in size.

Our previous data [12] show that elevated IOP causes activation of calcineurin in RGC, and the current data suggest that RGC degeneration follows activation of calcineurin even without elevated IOP. These results suggest that calcineurin activation may be a critical mediator of RGC degeneration in glaucoma, and raise the interesting possibility that low-tension glaucoma may be consequent to activation of calcineurin by a mechanism separate from elevation of IOP. Since calcineurin can be activated in various pathophysiological settings, calcineurin activation may lead to a common phenotype recognized clinically as glaucoma; if so, neuroprotective strategies targeting calcineurin or its downstream effectors may be useful in low- and high-tension glaucomatous degeneration.

### Summary

In sum, we observed a slowly progressive loss of dendritic complexity, somal shrinkage, and dendritic and axonal loss after transduction of RGC in normal mice with constitutively activated calcineurin. These changes closely reflected the phenotype seen in models of elevated intraocular pressure, in which activation of calcineurin had been postulated to contribute to RGC neurodegeneration. Taken together with previous data demonstrating the presence of activated calcineurin in the setting of surgically induced ocular hypertension and a genetic model of glaucoma [12], the current data using AAV-mediated local gene expression in RGC strongly support a role for calcineurin in glaucomatous damage. Therefore, blocking calcineurin activity is potentially a promising neuroprotective method in treating glaucoma.
